# Graphene Domain Signature of Raman Spectra of *sp*^2^ Amorphous Carbons

**DOI:** 10.3390/nano10102021

**Published:** 2020-10-14

**Authors:** Elena F. Sheka, Yevgeny A. Golubev, Nadezhda A. Popova

**Affiliations:** 1 Institute of Physical Researches and Technologies, Peoples’ Friendship University of Russia (RUDN University), Miklukho-Maklaya 6, 117198 Moscow, Russia; nad.3785@mail.ru; 2 Yushkin’s Institute of Geology, Komi Science Center, Ural Branch of RAS, Pervomayskaya 54, 167982 Syktyvkar, Russia; yevgenygolubev74@mail.ru

**Keywords:** *sp*^2^ amorphous carbons, amorphics with molecular structure, one-phonon and two-phonon Raman spectra, electrical anharmonicity, graphene domains

## Abstract

The standard D-G-2D pattern of Raman spectra of *sp*^2^ amorphous carbons is considered from the viewpoint of graphene domains presenting their basic structure units (BSUs) in terms of molecular spectroscopy. The molecular approximation allows connecting the characteristic D-G doublet spectra image of one-phonon spectra with a considerable dispersion of the C=C bond lengths within graphene domains, governed by size, heteroatom necklace of BSUs as well as BSUs packing. The interpretation of 2D two-phonon spectra reveals a particular role of electrical anharmonicity in the spectra formation and attributes this effect to a high degree of the electron density delocalization in graphene domains. A size-stimulated transition from molecular to quasi-particle phonon consideration of Raman spectra was experimentally traced, which allowed evaluation of a free path of optical phonons in graphene crystal.

## 1. Introduction

Raman scattering has become an overwhelming method of testing graphene-based solid carbons. A comprehend review [1] could be recommended as a guide to the vast literature on the subject while providing in-depth view on the physics when dealing with Raman spectroscopy of graphene-based solids. The subject concerns two issues, each of which is highly peculiar and complicated. The first is the theoretical background of Raman spectroscopy in the case, while the second is associated with a broad meaning of ‘graphene-based solid’ under study. Thus, phonons of perfect as well as defect and disordered crystals and molecular vibrations of nanosize samples are fundamentals of the first part forming the ground for two theoretical approaches, namely: solid-state and molecular ones (see Reviews [2,3] and references therein). On the other hand, graphene-based solids, ranging from very well organized three coordinated *sp*^2^ graphite, graphene, nanotubes, and nanoribbons, down to amorphous carbons, graphene quantum dots as well as various *sp*^3^-*sp*^2^ mixtures, and that is not all, present the rich content of the second part. Because of this, the modern Raman spectroscopy of graphene materials is a subtle art of a proper combination of the two components [1]. The problem is particularly sharp when the solid is definitely nanostructured, which raises an evident question of whether the phonon-based solid-state approach is applicable to the case or if its molecular counterpart should be at play. The standard view of Raman spectra (RSs) of graphene-based species with main structural features presented by dominating D, G, and 2D bands, have so far played a decisive role in the approach selecting. A deep similarity of the spectra for a large set of samples of defect and disordered crystals of graphite and graphene [4,5,6,7,8,9,10] has created a favorable basis for successful use and further improvement of the solid-state approach in its phonon-confinement format [11,12]. This format has legitimated the use of RSs parameters, such as position, bandwidth and intensity of the D, G, and 2D bands (particularly, largely exploited parameters IDIG, and $\Delta \omega_{D}$) for the determination of the confinement parameters, thus characterizing the carbon crystals nanostructuring (see a number of cases in [1] and references therein). The same standard appearance of the RSs of crystalline and non-crystalline graphene-based solids stimulated expansion of the phonon-confinement approach to amorphous carbons, graphene quantum dot, and other non-crystalline species (see [13,14,15,16]), thus presenting the latter now as the main stream in the consideration of the RSs of all graphene-based carbons “proposed by international consensus” [1].

Nevertheless, recent detailed investigations have clearly revealed a molecular nature of *sp*^2^ amorphous carbons (ACs) (amorphics for simplicity) [17,18], again raising the question about the choice between solid-state and molecular approach. Meeting this request, in the current article we intend to inspect which new information about *sp*^2^ ACs can be obtained from their spectra analysis based on the molecular spectroscopy background. To make the latter informative and convincing, we conducted a comparative study of RSs of a set of specially selected *sp*^2^ amorphics, the structure and chemical composition of which were carefully investigated. This study allowed us to make conclusion about the type of the amorphicity of *sp*^2^ solid carbon, supporting its molecular character [19], and to reveal the fundamental difference between the molecular ACs and nanostructured graphite and graphene crystals subjected to size confinement.

The paper is composed as follows. Section 2 presents the description of the species selected for the study. General comments concerning vibrational spectroscopy of amorphous carbons as well as main concepts of the molecular approach related to graphene molecules are considered in Section 3. Section 4 and Section 5 present the interpretation of the obtained RSs in the framework of molecular approximation, concerning one- and two-phonon fractures, respectively. The conclusion summarizes the main essentials received.

## 2. Amorphous Carbons under Study

**Figure 1:**
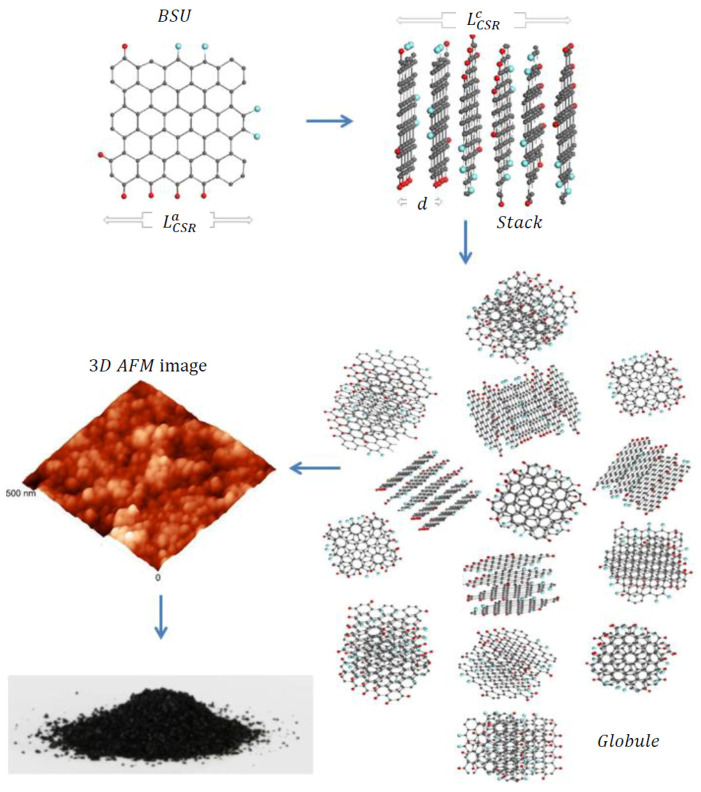
Schematic transformation from a single basic structure units (BSU) molecule (one of the BSU models C_66_O_4_H_6_ for shungite carbon [18]) to powdered solid amorphous carbon via BSU stacks and globule(s). The stacks consist of 4 to 7 BSU layers, differently oriented to each other. Planar view on a model globule composed of different stacks, with total linear dimensions of ~6 nm. Dark gray, red and blue balls depict carbon, hydrogen, and oxygen atoms, respectively. Three-dimensional atom-force microscope (AFM) image of globular structure of shungite carbon obtained with atomic force microscope NTEGRA Prima (NT-MDT, Zelenograd, Russia). The size distribution of the observed nanoscale particles peaked at 25 nm.

**Figure 2:**
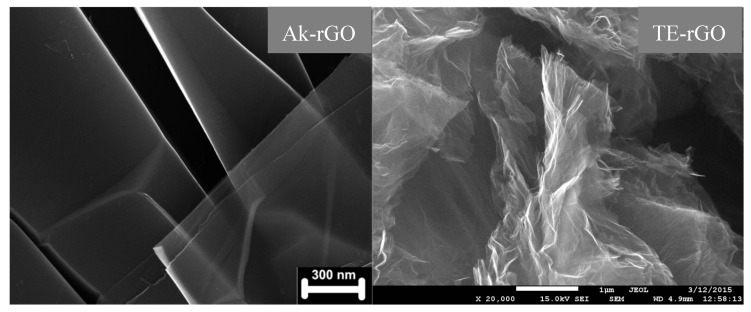
SEM images of technical graphenes Ak-rGO [27] and TE-rGO [28]. Adapted from Ref. [18].

Solid carbon belongs to covalent compounds, crystalline and amorphous states of which depend on the status of the relevant C–C bonds. The unique ability of carbon atoms to form three kinds of the bonds, differing by the electronic configurations of the atom valence electrons, provides the presence of *sp*^3^ and *sp*^2^ crystalline and amorphous allotropes that are strictly different concerning their structure and properties [19]. In the current study, our attention will be concentrated on *sp*^2^ ACs, which according to recent comparative studies [17,18], form a particular class of solids. As turned out, the general architecture of both natural and synthetic species is common and can be characterized as multilevel fractal one [20,21], albeit differing in details at each level. The first-level structure is similar in all the cases and is presented by basic structure units (BSUs). The higher-level one depends on the BSUs size. Thus, in the case of small-size natural amorphics, nanosize-thick stacks of nanosize BSUs present the second-level structure. These stacks form globules—a structure of the third level, characterized with pores of the first nanometers [22]. Further aggregation of globules leads to the formation of micro-nanosize agglomerates with pores of tens nm [22]. Figure 1 presents, schematically, the evolution of such an amorphic structure from a single BSU to macroscopic powder. Synthetic amorphics are characterized by a large dispersion of BSUs size from units to tens and/over first hundreds of nanometers. At the low-limit end of the dispersion, the amorphic structure is similar to that of natural species described above. At the high-limit end, the BSUs size does not prevent from BSUs packing in nanosize-thick stacks, while the latter laterally extended are further packed in a paper-like structure exemplified in Figure 2.

According to studies [17,18,23], BSUs of both natural and synthetic *sp*^2^ ACs present graphene molecules, which are based on a honeycomb fragments, or graphene domains. Obeying the general laws of chemistry of nanosize objects [24], the domains edge atoms are terminated by heteroatoms and/or atomic groups, including hydrogen, oxygen, nitrogen, sulfur, halogens mainly, making heteroatom necklaces. The molecules as a whole can be described by statistically averaged chemical formula (such as C_66_O_4_H_6_ (or C_6_O_0.36_H_0.55_ per one benzenoid unit) in the case of shungite carbon (a model BSU of which is shown in Figure 1), that corresponds to the chemical content of the sample obtained experimentally. Size, shape, and heteroatom necklace of BSUs, the latter includes terminating atoms and atomic groups at particular disposition in the molecule circumference, greatly vary, due to which each AC, classified usually by origin, is associated with a large class of specially framed graphene molecules. At the same time, despite the complexity of the overall fractal structure of *sp*^2^ ACs, precisely BSUs are the main players concerning the solids properties, thus stipulating molecular-structural approach for their description. Firstly, successfully applied to vibrational spectra of the solids provided with INS and DRIFT spectroscopies [18,25], in the current paper, the approach is extended over the Raman scattering. The set of the selected *sp*^2^ ACs of the highest-rank carbonization involved shungite carbon (ShC), anthraxolite (AnthX), and anthracite (AnthC), one chemically (Ak-rGO) and one thermal-shock (TE-rGO) reduced technical graphenes, as well as two industrially produced Sigma-Aldrich carbon blacks—699632 (CB632) and 699624 (CB624) (see detailed description of samples in [17,18]). The set is complemented with two graphites of the best quality from Botogol’sk deposit [26], characterized by mono- (mncr) and micronanocrystalline (μncr) structure. The structural and chemical data of the samples are summarized in Table 1 and Table 2. The data obtained earlier are supplemented in this study by the results of the X-ray diffraction and EDS measurements for the two graphites. Necessary to note, the data listed in both tables represent statistically averaged quantities. Actually, the positions of hydrogen and oxygen atoms in the BUS circumference, shown in Figure 2, can vary.

## 3. General Concepts of Molecular Approach Related to Graphene Molecules

Raman spectroscopy of *sp*^2^ ACs is generally aimed at determining their short-range structure [29,30]. However, the information obtained depends on theoretical motives laying the foundation of the spectra analysis. Thus, the solid-state approach sees the studied amorphics as carbon honeycomb fragments, which are presented by either flat or slightly curved [31] graphene sheets, with standard C=C interatomic space of 1.42 Å in size [1]. In contrast, a molecular approach suggests the consideration of these solids as honeycomb C=C covalent-bond compositions [32], with not fixed C=C bond lengths thus exhibiting their sensitivity to both environment and other conditions of the body’s production and storage as well making BSUs the main participants of the short-range structure. The next issue concerns the spectra internal content. In contrast to the IR-inactivity of molecular vibrational modes, associated with covalent homopolar bonds, caused by nil static moment [33], the electronic polarizability is quite favorable for these bonds. Respectively, IR photoabsorption reflects mainly the structure and chemical composition of the BSU heteroatom necklaces, and this has been recently successfully demonstrated by studying DRIFT spectra of the amorphics under consideration [18], while Ramat scattering draws the signature of the BSU graphene domains.

Vibrational spectrum of any polyatomic graphene molecule is multitudinous and multimode, due to which a certain simplification is needed to make it discussable. In the previous study [18], we suggested the spectrum of benzene molecule to be a benchmark. The list of benzene vibrational modes and their assignment are given in Table 3 [34]. According to symmetry rules, modes 1–10 are active in Raman scattering, while modes 11–20—in IR absorption. Evidently, any lowering of the molecule symmetry violates the double degeneracy and mixes the modes. Anyway, even with these limitations, both Raman and IR spectra of benzene molecule should be quite rich. However, in practice, both spectra of gaseous benzene are very simple and consist of small number of selected modes. Among the latter, only ν_1_ and ν_7_ modes form the pattern of the observed RS [35,36]. When the number of benzene rings increases, considerable growth of the vibrational modes occurs, and the spectra patterns remarkably change. As for linear chains of benzene rings, the governing role in the spectra, starting from naphthalene, goes to stretching mode ν_8_. The corresponding band is surrounded by satellites due to the violation of the *D_*6*h_* symmetry of benzene and the removal of the degeneracy of the initial mode, first weak in naphthalene and anthracene, and then comparable in intensity in tetracene and pentacene [37]. At the same time, a scattering of noticeable intensity in the region of modes ν_8_ and ν_19_ arises in the spectrum of the last two molecules. Therefore, the transformation of RS of benzene when going to pentacene consists in enhancing the role of C=C stretching vibrations, originated mainly from *e_*2*g_* ν_8_ and *e_*1*u_* ν_19_ benzene modes. The shape of the spectrum in this region is still rather complex.

A completely different feature is observed when going from polyacenes to polyaromatic hydrocarbons (PAHs) with two-dimensional (2D) *π* conjugated planar structure. The latter represent spatially extended compositions of benzenoid rings, forming restricted graphene domains framed by hydrogens. The first objects were PAHs C_78_H_32_ [38] and C_96_H_30_ [39], synthesized by Prof. Müllen’s team. Raman spectra of both molecules [40] drastically differ from the spectra of polyacenes, taking the shape of a characteristic D-G-2D-three-bands pattern, which is pretty similar to the spectra of *sp*^2^ ACs. Later on, the PAH set was enlarged including molecules of different shape, symmetry, and carbon content from C_24_H_12_ to C_114_H_30_ [41,42], whose RSs are of the same shape. Certainly, the similarity concerns the general pattern of the spectra, while IDIG, and $\Delta \omega_{D}$ parameters were quite individual. Nevertheless, it was experimentally shown that just the presence of graphene domains in the 2D planar structure of PAHs leads to the characteristic D-G-2D shape of the RSs, which does not depend on the size and symmetry of the PAH molecules. The band triplet covers D-G one-phonon and 2D two-phonon parts of the spectrum.

**Figure 3:**
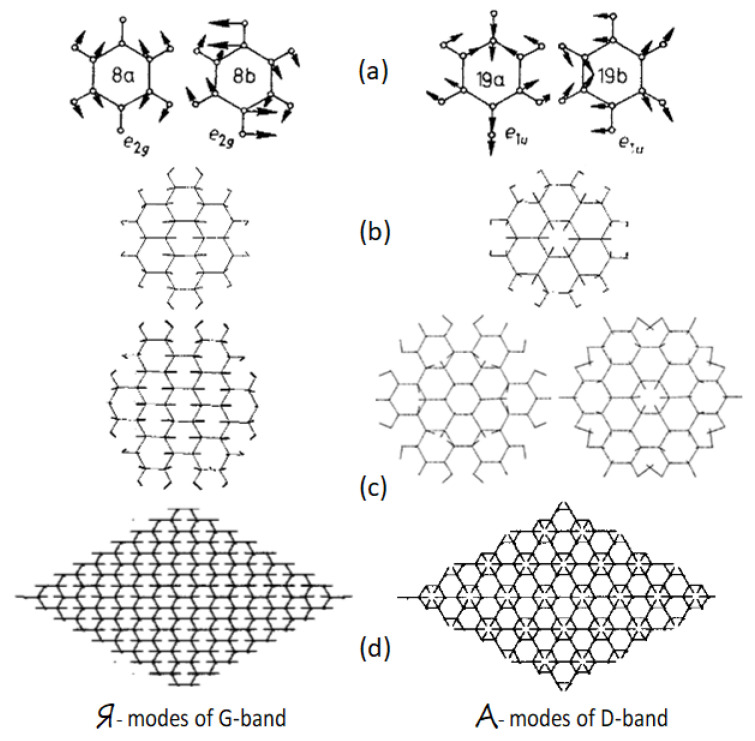
Vibrational forms of selected normal vibrations: (**a**) benzene; (**b**) coronene; (**c**) hexabenzocoronene; (**d**) a perfect 2D lattice of graphene. Composed of data from [34] and [40].

A profound theoretical analysis of one-phonon RSs performed by the Italian spectroscopists [40,41,42,43,44,45,46,47,48,49,50] allowed both to reveal the reasons for the discovered uniqueness of the PAHs RSs, and to establish their intimate connection with the spectra of graphite and/or graphene. It was found that the main contribution to the spectra intensity is made by C=C stretchings, due to which the observed D-G-2D set of bands is characteristic for the network of C=C bonds mainly. As for the stretchings themselves, the modes, which determine G band, are originated from the *e_*2*g_* vibration of benzene, while the modes responsible for D band come from the *e_*1*u_* vibration of the molecule (see Table 3). The modes individuality is caused by peculiarities of the form of their vibration, which visualizes shifting from the equilibrium each of the molecule atoms. As convincingly shown (see Figure 3), the vibrations of G band correspond mainly to simultaneous in-plane stretchings of all C=C bonds, while those related to D band concern both stretching and contraction of these bonds when carbon atoms move normally to them just imitating benzenoid ring breathing. The motion of carbon atoms has a collective character, for which the planar packing of benzenoid units is obviously highly preferable. In contrast, as seen in Figure 3a, the vibration forms of both *e_*2*g_* and *e_*1*u_* modes of benzene are local and differ much from collective forms of PAH molecules.

Polarization of molecules is highly sensitive to the vibration form. Actually, the quantity is generally described as [34]

(1) $$\alpha_{t}=\alpha +\sum_{i}{\left(\frac{\partial \alpha }{\partial Q_{i}}\right)}_{0}Q_{i}+\frac{1}{2!}\sum_{ik}{\left(\frac{\partial^{2}\alpha }{\partial Q_{i}\partial Q_{k}}\right)}_{0}Q_{i}Q_{k}+\cdots$$

where α is the polarizability in equilibrium position, *Q*’s are co-ordinates of individual normal vibrations, sets $\left\{Q_{i}\right\}$ and $\left\{Q_{k}\right\}$ present vibration forms of the *t*th vibration, and the subscripts _0_ of the differentials refer to the equilibrium position. The third and subsequent members of the power series represent the electrical anharmonicity. The intensity of one- and two-phonon Raman scattering is governed by the second and third terms, respectively. As seen from the equation, the vibration forms are directly involved into the intensity determination, differently for the Raman scattering of the first and second order.

Detailed consideration of the PAHs polarizability performed in the extensive study [40,41,42,43,44,45,46,47,48,49,50] showed that parallel-to-bond vibration forms, attributed by the authors to the type Я [42]), promote a steady intense one-phonon Raman signal G in all the studied molecules. The feature does not depend on the molecules’ symmetry and shape, and is caused by the $\frac{\partial \alpha }{\partial Q_{i}}$ derivatives, which all are positive. In contrast, normal-to-bond vibration forms of type A [42] promote, in this case, both positive and negative $\frac{\partial \alpha }{\partial Q_{i}}$ derivatives, due to which the intensity of D band is tightly connected with the “quality” of C=C bonds, which is reflected in the vibration forms. Therefore, if the bonds are identical, the contribution of A modes into the one-phonon signal is nil. In the opposite case, the signal is not nil and is bigger, the bigger the difference between the bonds [43]. This conclusion has been approved on a number of PAH molecules, whose RSs were obtained and analyzed [40,41,42,43,48,50], as well as by latest theoretical studies [32]. The extension of the above consideration over graphene crystal have revealed that *e_*2*g_* mode at *Г* point of the first Brillouin zone is typical Я-mode, while *A*’_1_ mode at *K* point clearly reveals A character (Figure 3d). The findings undoubtedly evidence a peculiar collective character of the graphene phonons caused by benzenoid-hexagon structure. Therefore, until the honeycomb packing of benzenoid units is not broken, the graphene molecules and/or supramolecules are characterized by the D-G patterned Raman spectra. Only a complete destruction of the sheet leads to this feature loosing, which was really observed for graphene after its extremely strong bombardment by Ar^+^ ions [51].

## 4. One-Phonon Raman Spectra of *sp*^2^ Amorphous Carbons

**Figure 4:**
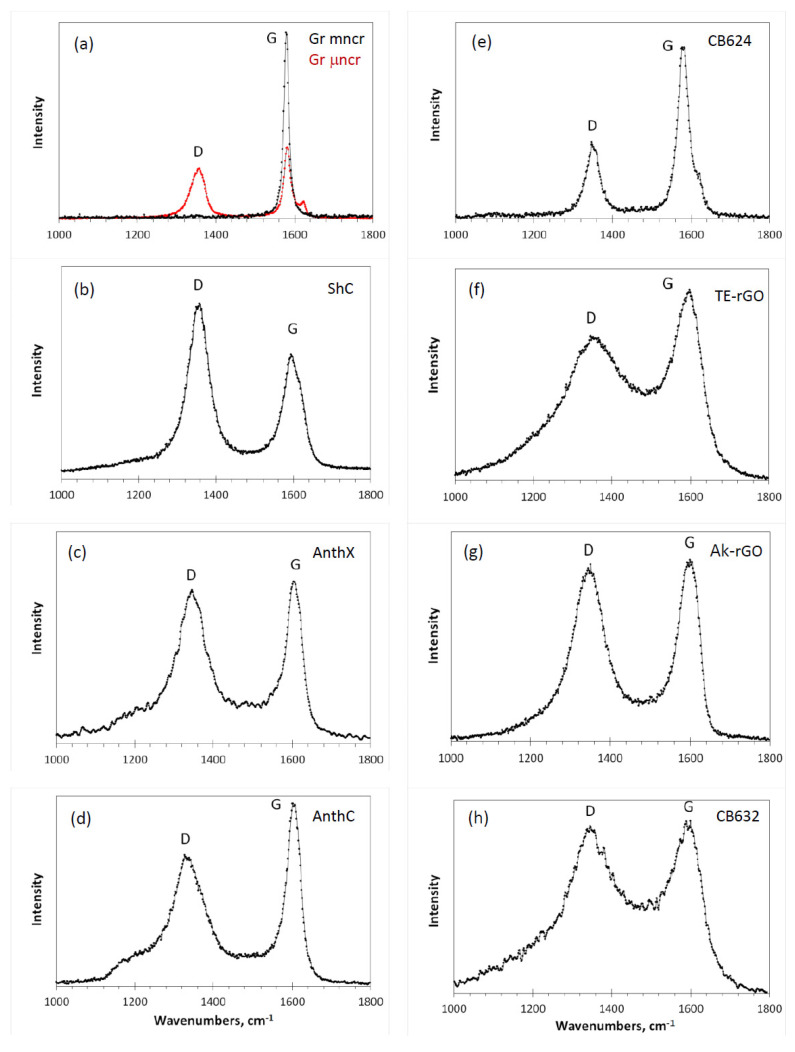
One-phonon Raman spectra of *sp*^2^ amorphous carbons at room temperature: shungite carbon (ShC), anthraxolite (AnthX), anthracite (AnthC), technical graphene TE-rGO [27] and Ak-rGO [26], carbon blacks CB632 and CB624, as well as mono- (mncr) and micronanocstructured (μncr) graphites, respectively; additional (**a**–**h**) marking facilitates the spectra description. Intensities are reported in arbitrary units.

Despite RSs of *sp*^2^, ACs were recorded countlessly (suffice to say that Raman scattering is an indispensable participant in testing each natural and synthetic graphene material), a detailed investigation of a set of different-origin representatives under the same level of knowledge about their structure and chemical content has not yet been performed. In the current study, nine products of such kind described in Section 2 were examined. Raman spectroscopy was carried out with a LabRam HR800 instrument (Horiba, Jobin Yvon, Villeneuve-d’Ascq, France) at room temperature. The system was equipped with an Olympus BX41 optical microscope (Olympus, Tokyo, Japan) and a Si-based CCD detector (1024 × 256 pixels) (New Jersey, USA). A 50× objective (working distance ~3 mm, numerical aperture 0.75) was used. Spectra were recorded in the 100–4000 cm^−^^1^ range, using a spectrometer grating of 600 g/mm, with a confocal hole size of 300 μm and a slit of 100 μm. External laser exciting radiation in the region 270–520 nm was used. The obtained RSs reveal the expected dependence on the radiation frequency typical for nanostructured graphite and or graphene (see [1] and references therein), as well as for PAHs [31]. Figure 4 presents a collection of RSs of the studied samples excited by the radiation of Ar^+^ laser (514.5 nm, 1.2 mW). This radiation is used in the majority of Raman scattering experiments, which makes it possible to compare the obtained spectra with the data available in the literature. Each spectrum in the figure is the result of three accumulations with a 10 s exposure.

The spectra are grouped in two columns related to natural (left) and synthetic (right) samples. Looking at this collection, we would like to start with the first features that concern fine-structured spectra located in the first row in the figure. Among the latter, there are two lineaments which require particular attention. The former is related to the RSs of graphites. A scrupulous analysis of the available RSs reveals that a single G-band-one-phonon spectrum is a rarity, once characteristic for “the best” graphites such as Madagascar flakes and Ticonderoga crystals [4], Ceylon graphites [52], Botogol’sk graphites [52,53], and some others. In contrast, in the predominant majority of cases, researchers are dealing with highly structured samples with a characteristic D-G doublet pattern of their RSs. However, even in these cases, graphite rocks are not structurally homogeneous, consisting of microscale monocrystalline (mncr) blocks surrounded with micronanocrystalline (μncr) graphite domains. Graphite spectra in Figure 4a exhibit these two constituents. Expectedly, BSUs of the studied graphites are of submicron lateral size (see $L_{CSR}^{a}$ data in Table 1), once terminated mainly by oxygen, the weight content of which constitutes generally ~1 wt% (see Table 2). Similarity of the spectrum of amorphous CB624 and that of the μncr graphite convincingly evidences that not only in the depth of the Earth core, but also in industrial reactors producing carbon black a significant graphitization of amorphous carbon may occur. D-G doublet of narrow band structure in μncr graphite and CB624 amorphic becomes the dominant feature of RSs of other studied ACs, both natural and engineered ones, but significantly broadened.

In the covalent-bond language of graphene molecules, the conversion of a single G band, corresponding to a solitaire optical G phonon of graphene crystals in a regular structure with strictly fixed C=C bond value at 1.42 Å, into a broadband D-G doublet of amorphous solid, is caused by two things. The first concerns an unavoidable generation of non-zero dispersion of the C=C bonds length (bond length dispersion (BLD) below), $\Delta \left\{l_{CC}\right\}$, in restricted graphene domains of amorphous solid. The second is a consequence of the graphene domain symmetry lowering, just resolving not only *e_*2*g_*, but *e_*1*u_* benzene-naming C=C stretchings discussed in the previous Section. Each of these groups covers a large number of C=C modes in polyatomic molecules like BSUs, thus splitting the total BLD into two sets, $\Delta {\left\{l_{CC}\right\}}_{e_{2g}}$ and $\Delta {\left\{l_{CC}\right\}}_{e_{1u}}$. Among the latter, the modes with a considerable extent of the Я and A character might contribute into the broad G and D bands.

**Figure 5:**
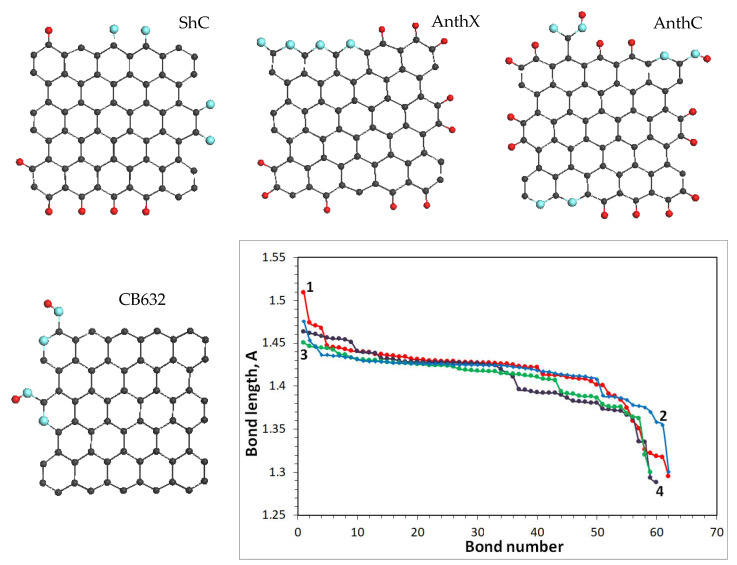
Equilibrium structure of BSU models related to shungite carbon (1), anthraxolite (2), anthracite (3), and carbon black CB632 (4). Distribution of C=C bond lengths of the model graphene cores. UHF AM1 calculations.

According to the vibrational dynamics of molecules, dispersion of any valence bond length $\Delta \left\{l_{i}\right\}$ is naturally transformed into that of force constants $\Delta \left\{f_{i}\right\}$ related to the relevant stretching. To evaluate the BLD value appertaining to the C=C stretchings of the studied amorphics BSUs, we perform quantum chemical calculations of the ground state structure of a set of BSU models related to commensurate small-size ACs with $L_{CSR}^{a}$ of 1.4–2.1 nm (see Table 1) [18]. All the models, shown in Figure 5, have the same graphene domain, consisting of 66 carbon atoms, of which only one (model 3), two (model 4), and four (model 2) atoms are substituted by oxygen. The structure set is complemented with the C=C bond length distribution related to each molecule. As seen in the figure, these distributions are quite similar for all the molecules, albeit remarkably varying in response to varying compositions of the models’ heteroatom necklaces, thus evidencing a different assortment of both bonds and vibrational frequencies of each amorphic. As seen in the figure, the C=C bond lengths cover the region from 1.5 Å to 1.3 Å in all the cases, thus determining the total dispersion of the bonds as $\Delta \left\{l_{CC}\right\}$ ≥ 0.2 Å. Unfortunately, because of a large number of vibrational modes, exact solution of the dynamical problem for the BSU molecules does not permit to distinguish $\Delta {\left\{l_{CC}\right\}}_{e_{2g}}$ and $\Delta {\left\{l_{CC}\right\}}_{e_{1u}}$ BLDs correctly [54]. So, we must limit ourselves by the discussion of the total value $\Delta \left\{l_{CC}\right\}$. Following Badger’s rule [55], vibrational frequency and bond length of C=C stretching are interconnected so that $f_{i}=3.0{\left(l_{i}+0.61\right)}^{-3}$ [56] for constant and length units in 10^2^ Nm^−1^ and 10^−10^ m, respectively. According to this relation, changing the length $l_{CC}$ from 1.5 Å to 1.3 Å corresponds to frequency growing from 1300 cm^−1^ to 1700 cm^−1^, which completely covers the frequency region of the D-G plottings for the studied solids.

The discussed above allows suggesting that the broadbandness of the D-G doublet structure of the RSs of *sp*^2^ ACs is caused by the length dispersion of the C=C bonds that configure the graphene domain structure of the relevant BSUs. Besides, the BSU mandatory termination by heteroatoms clearly exacerbates the latter, thus making it dependent on a BSU size and the relevant chemical necklace [54]. It is known as well [57,58,59] that the multilayer packing of graphene sheets significantly influences RS of each of them as well, pointing to the additional redistribution of C=C bonds in the layers. Therefore, Raman scattering features of *sp*^2^ ACs concern two first levels of their multilevel structure, namely, BSUs and stacks of them. Apparently, there are other factors affecting Raman scattering, such as, say, anharmonic contribution of which is expected to be quite strong in graphene molecules (see next Section). In what follows, we will analyze the obtained RSs in terms of the empirical triad involving size and chemical necklace of BSUs, as well as their packing.

Let us start from the RSs of natural amorphics in Figure 4b–d, BSUs of which are commensurate with PAH C_114_H_30_, RS of which was carefully analyzed [43]. As should be expected, the spectra of amorphics and PAH are well similar. The PAH spectrum consists of well isolated D and G bands with FWHM of 30 cm^−1^, due to which the gap between the band is well pronounced. The D band is accompanied with four clearly distinguished low-intense satellites around the main peak. The satellite surrounding is characteristic for the D band in all the studied PAHs [32,40,50]. As for amorphics spectra, the FWHM of both main D and G peaks is doubled comparing with that of the PAH, while the satellite escort of the PAH D band is transformed into marked pedestal of different fine structure for amorphics, significantly broadening the band in contrast to the G band.

In the light of the analytical triad described above, the spectrum of the PAH molecule contains information about the size of the graphene domain and the termination of the dangling bonds of its edge atoms with hydrogens. A detailed theoretical analysis of the spectra of similar PAHs was performed in terms of the totality of its C=C bonds in the presence of terminating hydrogen atoms [32], however, the contribution of the latter to the RS shape was not distinguished. Since, nevertheless, the spectrum of the C_114_H_30_ molecule consists of narrower bands than the spectra of the amorphics under consideration, it is quite reasonable to take it as the reference spectrum of a graphene domain of 1.5–2 nm in size. Respectively, the observed additional broadening of the amorphics RSs can be attributed to either heteroatom necklace of their BSUs or the BSUs packing.

Neutron and X-ray powder diffraction of shungite carbon and anthraxolite showed that the configurations of the solid stacks formed by the relevant BSUs were identical and the closest to the packing in graphite crystal (see [18] and references therein). Accordingly, these solids RSs broadening with respect to that of the PAH can be attributed to stronger effect of the BSU heteroatom necklace on the C=C BLD $\Delta \left\{l_{CC}\right\}$ in comparison with that of hydrogen atoms. Additional broadening can be caused by the variety of the necklace structure at fixed chemical content. Therefore, the difference of the ShC and AntX RSs in Figure 4b,c can be attributed to the different disturbance of the BLD $\Delta \left\{l_{CC}\right\}$ by different heteroatom necklaces, which is supported with the bond length distributions shown in Figure 5. Similarly, the difference of the AnthC RS in Figure 4d from the spectra of ShC and AntX can be attributed to the changes in the relevant necklace. Nevertheless, the structure study of the latter revealed much stronger deviation of BSUs packing from the graphite one [18] due to which the RS shape, particularly in the D band region, may additionally reflect the changing of BSUs packing in this case towards a turbostratic one [60].

To the most extent, the influence of the turbostratic packing is seen when comparing the RS of carbon black CB632 in Figure 4h, with the spectra of natural amorphics discussed above. As seen in the figure, the CB632 spectrum drastically differs from those of natural species despite all the BSUs are commensurate and the influence of the BSU heteroatom necklace is also comparable (see Figure 5). At the same time, structural data clearly evidence the turbostratic packing of the BSUs in this case (see [25] for details), which causes such a strong broadening of the spectrum thus manifesting the effect of neighboring layer on the C=C BLD in each individual layer.

Based on the conclusions made when analyzing RSs of small-size natural amorphics and carbon black CB632, we can proceed with the interpretation of RSs of technical graphenes Ak-rGO and TE-rGO, as well as carbon black CB624, related to large-size amorphics. As seen in Table 1, amorphous technical graphenes are characterized by large BSUs of submicron size, bigger than that one of carbon black CB624. Nevertheless, their RSs in Figure 4f,g differ drastically from the spectrum of the latter in Figure 4e, clearly evidencing strong disordering and large BLD $\Delta \left\{l_{CC}\right\}$ in the relevant graphene domains. It is important to note that the two spectra strongly differ in between as well. At the same time, the Ak-rGO spectrum is similar to that of AntX, while the same can be said about the spectra pair of TE-rGO and CB632. In both cases, the similarity is observed despite more than one order of magnitude difference in the BSUs size.

**Figure 6:**
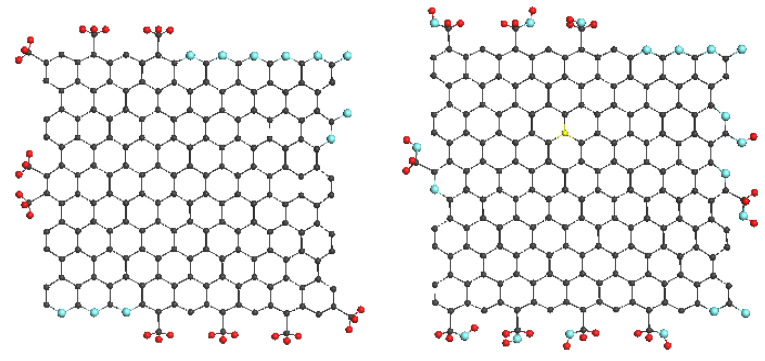
Molecular models of basic structure units of technical graphenes Ak-rGO (**left**) and TE-rGO (**right**). Light gray, red and black circles mark carbon, oxygen and hydrogen atoms.

Discussing RSs of natural amorphics, we referred to the spectrum of the PAH C_114_H_30_ as the reference related to small-size graphene domains. In the case of large-size domains, evidently, the spectrum of CB624 in Figure 4e can play the role. The comparison of the Ak-rGO spectrum with the reference reveals the doubling of FWHMs for both D and G bands at maintaining the general D-G appearance of the spectra as a whole. The feature is similar to that one resulted from the comparison of the PAH C_114_H_30_ and AnthX spectra. Since the additional broadening of the AnthX spectrum, which is similar to that of Ak-rGO, we attributed to the changing of the BLD $\Delta \left\{l_{CC}\right\}$ caused by complicated heteroatom necklace of the relevant BSUs, it is reasonable to replicate this conclusion with respect to the Ak-rGO–CB624 pair of spectra. The model necklace related to Ak-rGO amorphic, suggested on the basis of extended neutron scattering and DRIFT studies [18], is shown in Figure 6 (left). As seen in the figure, it is quite cumbersome, while causing the broadening comparable with that of AnthX. Additionally, despite the fact that the necklace is much more complex with respect to that of AnthX, the D band in the RS of Ak-rGO is more symmetric without traces of the satellite surrounding structure. Similarly, the D band in the RS of CB624 in Figure 4e is symmetric, which apparently is resulted from the large size of the relevant BSUs. In contrast to Ak-rGO, RS of the TE-rGO is drastically different, despite the commensurate BSU and similarity of the complex chemical necklace (see Figure 6 (right)). A close resemblance of RSs of this amorphic and CB632, discussed earlier, leads to the conclusion that in both cases, a particular packing of the BSU layers is responsible for the spectra broadening. Actually, as in the case of CB632, neutron and X-ray powder diffraction reveals the turbostratic packing of paper-like sheets of TE-rGO [28], the consequences of which are clearly visible in Figure 2 when comparing the external view of the Ak-rGO and TE-rGO solids.

**Figure 7:**
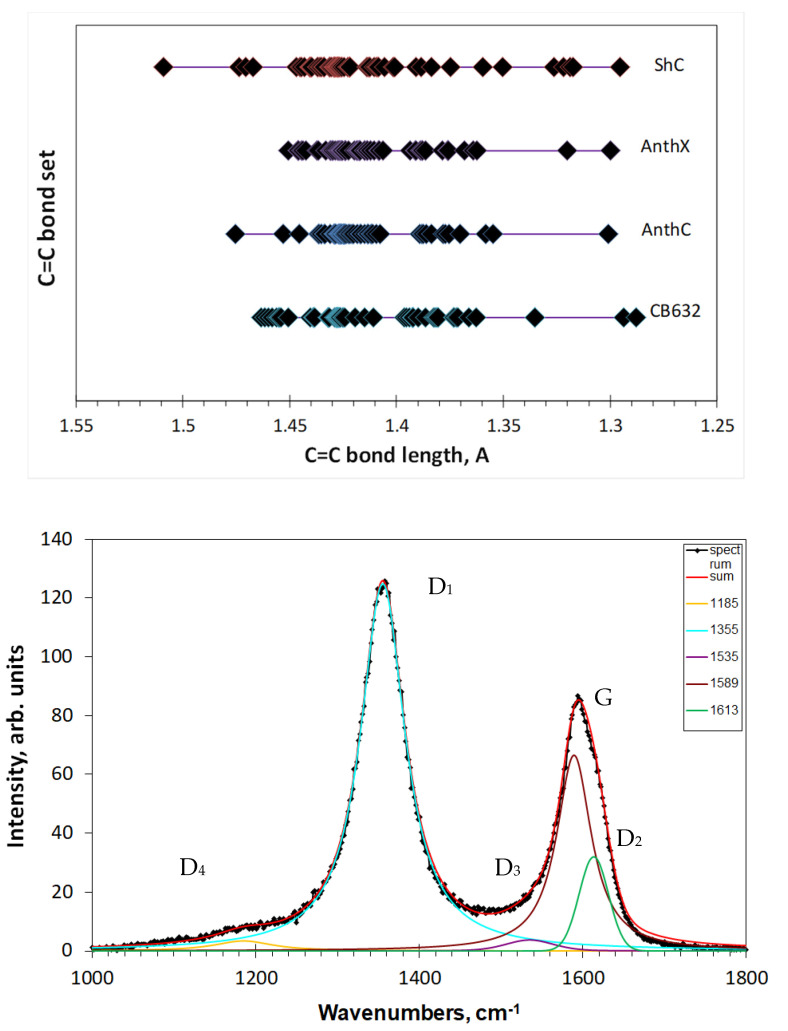
C=C bond sets of the model graphene domains (**top**) and deconvoluted Raman spectrum of shungite carbon (**bottom**) (see text).

The analysis performed above convincingly shows that the C=C bond length distributions are responsible for the complicated broadband structure of the observed D-G spectra of *sp*^2^ ACs. As seen in Figure 5, the distribution is not homogeneous over the length scale and can be grouped. Evidently, grouping of C=C bonds in the studied amorphics causes a similar response of the C=C stretchings frequencies, thus laying the foundation of ‘multiband’ origin of their broadband D-G RSs. To stress the attention on such grouping, Figure 7 (top) presents the bond length distributions, shown for exemplary models in Figure 5, in a different way. As seen in the figure, C=C bonds actually form distinguished groups. It is natural to expect that in the real RSs each of these groups should be associated with a relatively narrow band, because of which the observed spectrum represents a convolution of such bands.

Intuitively, the multiband character of the D-G spectra was taken into account and adopted by spectroscopists from the first studies of Raman scattering by amorphous carbon and graphites (see, for example, [53,61] and references therein). Standard programs of spectra deconvolution, such as LabSpec 5.39 in the current study, were widely used for experimental spectra decomposition. A typical example of such treatment is presented in Figure 7 (bottom). The set of D1–D4 bands complemented with G band presents the usual basis for decomposing. Quite narrow spectral region and governing role of D1 and G bands provide a rather small dispersion in the maximum positions and FWHMs of D2–D4 bands, thus giving a possibility to use the D1–D4 and G bands features when comparing RSs of different samples. Until now, it has been a comfortable formal language only facilitating the spectra description, while any changing of the D-G spectrum shape directly exhibits the reconstruction of the C=C bonds sets of the samples BSUs graphene domain.

Concluding the analysis of one-phonon D-G spectra, one should focus on the connection of widely used parameter IDIG with the real size of graphene domains. The advantage of the current study is the availability of independent data on the size of the studied BSUs obtained previously [17,18] and presented in Table 1. Based on these data, we come to unexpected results. So, for natural amorphics (spectra in Figure 4b–d), the BSU sizes of which are approximately the same, the IDIG doubles. The parameter for CB632 (Figure 4h) of practically the same lateral size is close to that for AnthX, but is different from both ShC and AnthC. The same can be said about the parameters for spectra CB624, TE-rGO, and Ak-rGO (Figure 4e–g) with commensurate size parameters. Thus, the parameter IDIG can hardly be considered as an indicator of the graphene domains size in this case. The same conclusion was reached by the Italian researchers on the analysis of the D-G spectra of PAHs [42]. At the same time, on the experimental field of Raman spectroscopy of materials with elements of a graphene structure, there is a widespread belief in the unambiguous relationship of this parameter with the size of graphene domains. At the time, this statement was confirmed by joint studies of the structure (X-ray diffraction) and Raman spectra with the determination of the ratio for nanostructured graphite and graphene crystals (see [6,7,8,9] and references therein). The first IDIGvia La relation proposed by Tuinstra and Koenig [4], gradually changed its appearance, supplemented by new parameters. The largest recognition in practice has received the relation suggested by Conçado et al. [62]. Based on a spatial confinement model applied to phonons in disordered graphene-based carbons [11,12], this approach works quite satisfactorily in the cases of microstructured solids. Encouraged by this success, practitioners of Raman spectroscopy began to use these relationships in the case of nanostructured objects as well (see [61] and references therein), for most of which there are no independent structural data. Actually, only in this work such data are presented, which allows us to call into question the validity of using the ratio to determine $L_{a}$. Table 4 shows the $L_{a}$ data obtained by processing the spectra shown in Figure 4 using the relations IDIGvia La from [4,62].

As seen from the table, according to X-ray data, $L_{a}$ should be the largest for shungites among the ShC-AnthX-AnthC-CB632 group. In contrast to these expectations, the value, obtained from Raman spectra, turns out to be the smallest, so the inverse X-ray dependence is observed. As seen from the table, the wished correspondence violates when the domain size is of the first tens of nanometers as well. In the present case, the situation does not seem unexpected, since, as was shown earlier, not only the size of graphene domain of the amorphics BSUs, but also the relevant heteroatom necklaces, as well as the BSUs packing determine the RS intensity distribution between its constituent bands.

## 5. Two-Phonon Raman Spectra of *sp*^2^ Amorphous Carbons

Second-order RSs of graphene materials have a long and rich history. Initially discovered in different graphite materials in the form of doublet of bands at ~2720 cm^−1^ (strong) and 3248 cm^−1^ (very weak) [63,64], designated as G′(then 2D) and 2G, respectively, this region of the spectrum, demonstrating interesting properties, since that has been actively analyzed [65,66,67,68] (references are representative rather than exhaustive). Referring the reader to an excellent review of the state of art in this area [6], we will focus only on two features of the 2D band, which are important for further discussion. The first concerns the fact that there are no bands in the one-phonon spectrum of ideal graphite [64] and graphene [66] crystals, whose overtones or composites can be assigned as the 2D band. Only in graphite and/or graphene materials, which are obviously devoid of an ideal structure, the source of the overtone is attributed to the band D. This feature of “no one-phonon source” is tightly connected with the origin of the two-phonon spectrum of graphene materials. According to the fundamentals of Raman scattering [34], anharmonic dependence on normal coordinated of both vibrational and electronic energies (so called mechanic and electric anharmonicities) leads to the appearance of IR and Raman vibrational spectra beyond one-phonon. Mechanic anharmonicity is described with the third derivatives of potential energy, while the electric one results from the second derivatives of the object polarizability in Equation (1). The two features influence the band-shape of the two-phonon spectra differently. Thus, the inconsistency of “no one-phonon source” and two-phonon spectra found in the case of RS of graphene crystals convincingly evidences a predominant role of the electric anharmonicity (*el*-anharmonicity below).

The second peculiarity of the 2D band concerns the surprising variability of its intensity with respect to the G band. Thus, in graphite crystal, the ratio of the total intensities I2DIG is ~1; in graphene crystal, it is equal to ~6 [67]; and in graphene whiskers, it exceeds 13 [66]. Such large values and sharp fluctuations of the intensity, as well as the observation of 2D bands in graphite whiskers with an ideal crystal structure of not only intense 2D band, but also a broad high order RS located in the high-frequency region up to 7000 cm^−1^, indicate an exceptionally big role for *el*-anharmonicity in this case. We are not aware of other examples of such a striking effect. Apparently, this property should be added to the treasure-box of graphene materials uniqueness.

Despite the huge number of publications concerning RS of graphene materials and the 2D band, in particular, the relationship of the 2D band with *el*-anharmonicity has not been yet considered. At the same time, modern vibrational spectroscopy attaches great importance to both mechanical and electrical anharmonicity, a joint participation of which provides good agreement between the experimental and calculated spectral data without fitting parameters [69,70,71]. Currently, this new approach can be applied to mid-size molecules such as thiophene or naphthalene, but the work on computational modules continues, so that graphene molecules of the first nm in size will be apparently able to be considered in the near future. Nevertheless, already obtained data on the quantitative accounting of anharmonicity in small molecules allow suggesting that the above-described behavior of the 2D band intensity in ideal graphite and/or graphene crystals at a qualitative level is quite expected, if to assume the anharmonic behavior of the vibrational and electronic spectra of these structures to be peculiar. We dare to suggest that the highly delocalized character of the electron density of the graphene crystal and domains [72] contributes to such a pronounced anharmonicity. Moreover, the role of this feature in mechanical effect is evidently not direct. In contrast, the second derivatives of polarizability are directly determined by the state of the electronic system, which, possibly, determines the special role of *el*-anharmonicity in graphene. When the article was already written, it became known [73] that the intensity of G and 2D bands in graphene depends on the laser intensity in opposite way—it increases (G) and decreases (2D) with increasing the power, respectively. The authors explained the feature with asymmetric Fermi–Dirac distribution at the different optically resonant states contributing to Raman scattering stimulated with high electronic temperatures reached for pulsed laser excitation. Evidently, the distribution is tightly interrelated with the delocalized character of the electronic state.

**Figure 8:**
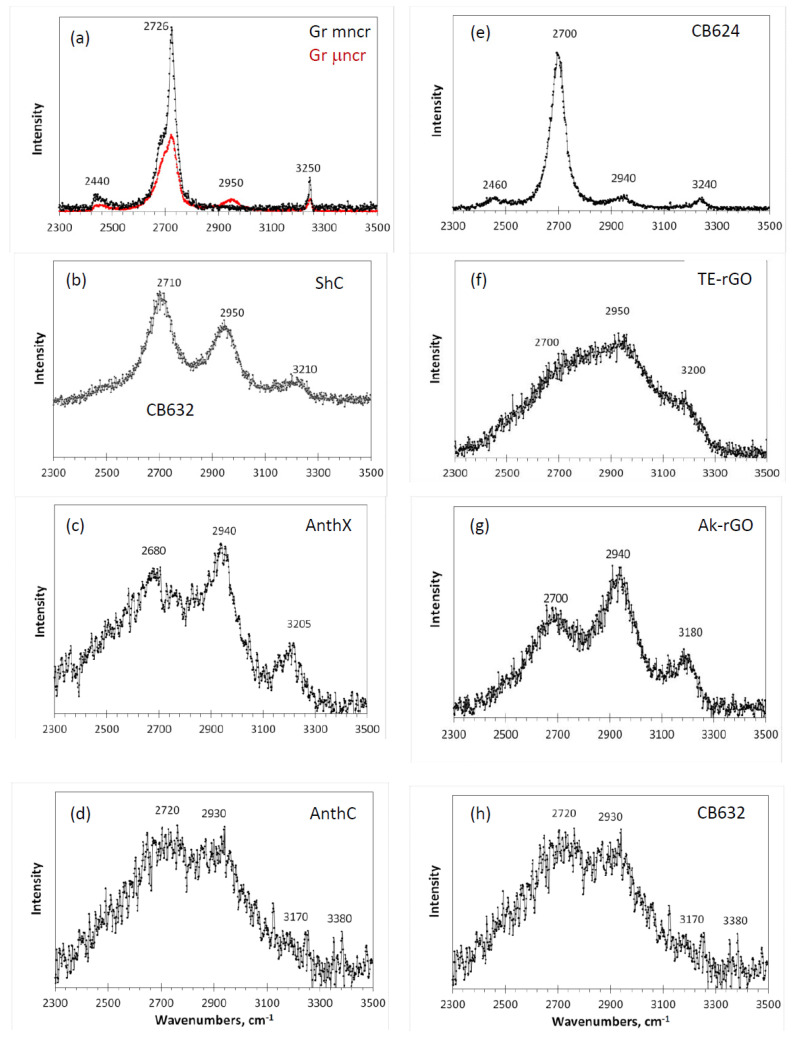
Two-phonon Raman spectra of *sp*^2^ amorphous carbons. Sample and (**a**–**h**) markings are the same as in Figure 4.

Delocalization of electron density is not a prerogative of crystalline structure, but lays the foundation of specific properties of graphene molecules as well [74]. Supposing its particular role in RSs of the species, the observation of the second-order RS in such molecules as PHAs becomes obvious. It is this kind of spectrum that was recorded in the case of two PAH molecules [40] (C_78_ and C_96_ [43]). Looking at the spectra from this standpoint, we see that, actually, both of them consist of the doublet of well-defined and narrow bands G and D located at 1603 and 1316 cm^−1^, accompanied by a rather intense broad band with weakly expressed maxima at ~2610, ~2835, and ~2910 cm^−1^. The first and last frequency markers are in good agreement with the frequencies of 2D and D + G bands. The appearance of the second maximum is obviously associated with the combination bands whose sources in the one-phonon spectrum have yet to be determined, thus supporting the *el*-anharmonicity origin of the spectra. In general, the band-shape of the two-phonon RSs of both molecules is similar to those of the studied *sp*^2^ ACs shown in Figure 8. Similar to one-phonon RSs, the spectra are grouped in two columns related to natural (left) and synthetic (right) samples.

As seen in the figure, a characteristic four-component structure, clearly distinguished in the spectra of two graphites (Figure 8a) and amorphous carbon black CB624 (Figure 8e), can be traced in the spectra of all other amorphics. Three frequency markers at ~2700, ~2940, and ~3200 cm^−1^, convincingly attributed to 2D, D + G, and 2G combinations, are steadily observed in all the spectra. The fourth marker at 2440 cm^−1^, whose assignment remains unclear, is hidden in the low-frequency tails. It is important to note that this marker involves at least one fundamental mode with frequency less than that of the lowest D mode. This mode has never been observed in the one-phonon spectra. This “no- one-phonon-source” feature is one more argument in favor of the *el*-anharmonic origin of the second-order Raman scattering of the studied *sp*^2^ ACs.

Among the studied amorphics, the spectrum of black carbon CB624 occupies a central place. Thus, its close similarity with the spectrum of μncr graphite indicates that, despite the obvious difference in size of the short-range order region in the graphite and amorphic BSU, in both cases, we are dealing with well-ordered structures making it possible to consider the spectra band-shape in the quasi-particle phonon approximation (widely reviewed in [67,68]). As known from the solid-state physics, quasi-particle description, based on the conservation of translational symmetry, is applicable with respect to size-confined bodies when the size of the latter exceeds a critical value characteristic to the particle under consideration. In the case of phonons, the phonon free path determines this critical size (see the discussion of the issue for amorphics with molecular structure in [75,76]). The crystal-like band-shape of the RS of the carbon black CB624 under consideration evidences that the free path of the high frequency optical phonons in graphene crystal is ~15 nm, as follows from Table 1. This conclusion is fully supported by the crystal-like behavior of the one-phonon spectra of this solid shown in Figure 4.

Since the theory of high-order RSs of large molecules is still being formed, a rigorous interpretation of their spectra, similar to proposed for one-phonon spectra in terms of molecular dynamics [5] is difficult. In connection with that, we propose to look at the spectra in Figure 8 from the viewpoint of analytical triad: the size and heteroatom necklace of the relevant BSUs, as well as the BSUs package—as it was done with the one-phonon spectra in the previous section. As seen in the figure, the spectra form two distinguished groups, which join spectra of natural amorphics and Ak-rGO, on the one hand, and spectra of TE-rGO and CB632, on the other. This grouping replicates that one of one-phonon spectra in Figure 4, clearly evidencing common reasons providing a significant likeness of the spectra within the group and a pronounced dislikeness between the groups. Obviously, the discussed features are in line with the concept on the governing role of the C=C BLD, thus allowing one to attribute the RS shape of the first group solids to the effect of heteroatom necklace of individual BSUs, while that one of the second group—to the BSUs turbostratic packing effect. Evidently, not the C=C BLD $\Delta \left\{l_{CC}\right\}$ itself, as in the case of one-phonon spectra, but its manifestation through the *el*-anharmonic action is responsible for the 2D spectra broadening. Despite the theory that *el*-anharmonic RS [69,70,71] has not so far been allowed to formulate general regularities that govern the selection of particular modes for two-phonon spectra, it is evident that the action concerns a group of modes and, when the modes pool is large, this leads to a significant broadening of the two-phonon bandshape. It should be noted that the two-phonon spectra turned out to be more sensitive to the triad components than one-phonon ones.

Concluding the analysis, one more thing should be mentioned. The frequency range of the studied two-phonon spectra coincides with the region of characteristic group frequencies (GFs) [77,78], related to C-H stretching vibrations. Since, as seen in Table 2, all the studied amorphics, besides carbon blacks, are significantly hydrogenated, we tried to find the evidence of the hydrogen presence. However, none of the features related to the spectra observed can be attributed to characteristic GFs, such as 3050 cm^−1^ related to methine groups [34] of natural amorphics, as well as 2870–2970 cm^−1^ and 2920–2980 cm^−1^ of methylene and methyl groups [34] of technical graphenes TE-rGO and Ak-rGO, respectively. If hidden inside the broad bands, they might be revealed by a particular technique to be developed.

## 6. Conclusions

Long-time and numerous studies of amorphous solids have led researchers to the conclusion that a confident interpretation of their Raman spectra is inseparable from the consideration of the nature and type of the solids amorphization. Previous structural and analytical studies of amorphous carbons were analyzed from this viewpoint, in course of which it was subsequently established that the solids should be attributed to molecular amorphics of a new type of amorphicity, which can be called the enforced fragmentation of honeycomb canvas [19]. Chemical reactions occurring at the fragment edges are suggested to be one of the most important causes of the fragmentation stabilization. Thus originated fragments, becoming the basic structural units (BSUs) of AC, are of a particular kind, presenting size-restricted graphene domains in the halo of heteroatom necklaces. The weak wdW interaction between the BUSs makes them responsible for the numerous properties of solids, including their IR absorption and Raman scattering.

Raman spectra of *sp*^2^ ACs are considered at the molecular level, which is perfectly suitable to the case. The molecular approximation is confirmed by a detail similarity of the spectra of the studied solids and polyaromatic hydrocarbons examined earlier. The similarity convincingly evidences a governing role of the extended graphene domains, of both BSUs and PAHs, due to which a standard D-G-2D three-band image of the *sp*^2^ ACs Raman spectra presents the graphene domain signature and remains until the domain structure is fully destroyed. The theory of Raman spectra of PAHs molecules, proposed by a team of Italian authors and confirmed in a number of convincing computational experiments, laid for the approach foundation. In the current study, it is proposed to extend the conceptual results of the theory to the case of the BUSs of the studied amorphics and to supplement the analysis of their Raman spectra by the concept of the governing role of the C=C bond length dispersion (BLD). The issue explains the appearance of the D band as well as the spectra broadening as a whole. Besides, the available structure and chemical content data of the studied amorphics allowed suggesting an analytical triad: the size and heteroatom necklace of the BSUs, as well as the BSUs packing—as an additional tool for the *sp*^2^ ACs spectra interpretation. This approach was applied to the interpretation of the one-phonon (D-G) and two-phonon (2D) spectra of the studied solids separately.

*One-phonon spectra*. Evaluated computationally, the C=C BLD for the studied amorphics constitutes ~0.2 Å attributing to the change of bond length from 1.5 Å to 1.3 Å. The corresponding dispersion of the C=C stretching frequencies is of ~400 cm^−1^ from 1300 cm^−1^ to 1700 cm^−1^, thus covering the main spectral region of the amorphics RSs. The distribution of the bonds within the dispersion region depends on the BSU size, the composition of heteroatom necklace, and the BSUs multilayer structure. In light of this triad, the shape of the D-G spectra of natural amorphics and chemically reduced technical graphene is provided with the BSU size, over which a significant heteroatom-necklace effect is put on. In addition, the spectra of temperature-shock exfoliated technical carbon and one of the engineered carbon blacks exhibit a marked contribution of the BSU packing effect caused by a turbostratic composition of the BSU stacks.

*Two-phonon spectra*. The C=C BLD concept allows explanation of the broadband 2D spectra of the studied solids as well, albeit not so straightforwardly as in the case of one-phonon ones since the spectra origin is provided with the electric anharmonicity of the BSU molecules. Nevertheless, size, heteroatom necklace, and packing effects are definitely exhibited in this case as well, thus making D-G-2D spectra of *sp*^2^ ACs to be a highly characteristic graphene domain signature. It is this last circumstance that connects details of the Raman spectra of the AC BSUs in question with the history of the origin, production, and storage of the studied solids.

## Figures and Tables

**Table 1:** Structural parameters of amorphous carbons ^1^.

Samples	Interlayer Distance, d (Å)	$L_{CSR}^{c},\;\mathrm{nm}$	Number of BSU Layers	$L_{CSR}^{a},\;\mathrm{nm}$	Ref.
mncr Gr ^2^	3.35(X)	105	313	550	this work
μncr Gr ^2^	3.35(X)	49	146	184	this work
ShC	3.47(N); 3.48(X)	2,5(N); 2.0(X)	7(N); 5–6(X)	2.1(X)	[17]
AnthX	3.47(N); 3.47(X)	2.5(N); 1.9(X)	7(N); 5–6(X)	1.6(X)	[17]
AnthC (Donetsk)	3.50(X)	2.2(X)	5–6(X)	2.1(X)	[17]
CB632	3.57(N); 3.58(X)	2.2(N); 1.6(X)	6(N); 4–5(X)	1.4(X)	[17]
CB624 ^3^	3.40(N); 3.45(X)	7.8(N); 6(X)	23(N); 17(X)	14.6	[17]
Ak-rGO	3.50(N)	2.4 (N)	7(N)	>20(N) ^4^	[27]
TE-rGO	3.36(N)	2.9 (N)	8(N)	>20(N) ^4^	[28]

^1^ Notations (N) and (X) indicate data obtained by neutron and X-ray diffraction, respectively. ^2^ The data are obtained by the treatment of (002) and (110) reflexes using Scherrer’s relation *L_CSR_* = *K·*λ/*B·*cos*Θ*. Here λ is the X-ray radiation wavelength (CuK_α_) 0.154 nm, *Θ* is the position of the (110) ($L_{CSR}^{a}$) and (002) ($L_{CSR}^{c}$) peaks, *B* is the half-height width of the peak in 2*Θ* (rad) units, and constant *K* constitutes 0.9 and 1.84 for reflexes (002) and (110), respectively, in the approximation of disk-shaped particles. ^3^ X-ray data were corrected in the current study. ^4^ $L_{CSR}^{a}$ = 20 nm means the low limit of the value accessible for the measurements performed.

**Table 2:** Chemical content of amorphous carbons.

Samples	Elemental Analysis, wt%						XPS Analysis, at%			
	C	H	N	O	S	Ref.	C	O	Minor Impurities	Ref.
mncr Gr ^1^	99.0	-	-	1.0	-	this work				
μncr Gr ^1^	98.9	-	-	1.1	-	this work				
ShC	94.44	0.63	0.88	4.28	1.11	[17]	92.05	6.73	**S**—0.92; **Si**—0.20; **N**—0.10	[17]
AnthX	94.01	1.11	0.86	2.66	1.36	[17]	92.83	6.00	**S**—0.85; **Si**—0.25; **N**—0.07	[17]
AnthC	90.53	1.43	0.74	6.44	0.89	[18]	92.94	6.61	**Cl**—0.11; **S**—0.34	[18]
TE-rGO	84.51	1.0	0.01	13.5	1.0	[18]	86.77	10.91	**F**—077; **S**—0.86; **Si**—0.70	[18]
Aк-rGO	89.67	0.96	0.01	8.98	0.39	[18]	94.57	5.28	**S**—0.16	[18]
CB624	99.67	0.18	0	0.15	-	[17]	95.01	4.52	**Si**—0.46	[17]
CB632	97.94	0.32	0.04	1.66	0.68	[17]	93.32	6.02	**Si**—0.66	[17]

^1^ EDS measurements.

**Table 3:** Vibrational modes of benzene molecule (composed of data from [34]).

Mode Number	Symmetry Assignment	Wavenumbers cm^−1^	Covalent-Bond Attribution
1	*a*_1*g*_	993	Breathing
2		3073	C–H stretching in phase
3	*a*_2*g*_	1350	C–H in-plane in-phase bending
4	*b*_2*g*_	707	C–C–C puckering
5		990	C–H out-of-plane trigonal bending
6	*e*_2*g*_	606	C–C–C in-plane bending
7		3056	C–H stretching
8		1599	C–C stretching
9		1178	C–H in-plane bending
10	*e*_1*g*_	846	C–H out-of-plane C_6_ libration
11	*a*_2*u*_	673	C–H out-of-plane in-phase bending
12	*b*_1*u*_	1010	C–C–C trigonal bending
13		3057	C–H trigonal bending
14	*b*_2*u*_	1309	C–C stretching (Kekulé)
15		1146	C–H in-plane trigonal bending
16	*e*_2*u*_	404	C–C–C out-of-plane bending
17		967	C–H out-of-plane bending
18	*e*_1*u*_	1037	C–H in-plane bending
19		1482	C–C stretching
20a		3064	C–H stretching

**Table 4:** Spectral characteristics of one-phonon Raman spectra of *sp*^2^ amorphous carbons and a comparison of the data-treated and independently obtained size of graphene domains $L_{a}$.

Samples	FWHM G, cm^−1^	FWHM D, cm^−1^	IDIG 1	*L_a_* According to Cançado et al. [62]	*L_a_* According to Tuinstra and Koenig [4]	*L_a_* XRD ^2^
ShC ^3^	40–45	50–70	2.2–2.7	6.5–7	1.5–2	2.1
AnthX ^4^	40–47	90–110	1.3–2.0	8–11	2–3.3	1.6
AnthC	40	100	2.0	8.3	2.1	2.1
CB632	85	110	1.1	15	3.9	1.4
CB624	34	45	0.6	29	7.6	14.6
TE-rGO	76	130	1.05	16	4.27	>20
AK-rGO	56	93	2.2	7.5	2	>20

^1^ Integral intensities are considered. ^2^ See sources of the data in Table 1. ^3^ Data average over 7 samples [53]. ^4^ Data averaged over 8 samples [53].
